# Dieta Rica em Gordura e Coração: Qual é o Real Impacto do Treinamento com Exercícios de Resistência?

**DOI:** 10.36660/abc.20240091

**Published:** 2024-04-22

**Authors:** André Rodrigues Lourenço Dias, Andrey Jorge Serra

**Affiliations:** 1 Universidade Federal de São Paulo Disciplina de Cardiologia Laboratório de Fisiologia Cardíaca São Paulo SP Brasil Universidade Federal de São Paulo, Disciplina de Cardiologia, Laboratório de Fisiologia Cardíaca, São Paulo, SP – Brasil

**Keywords:** Coração/fisiopatologia, Gorduras/metabolismo, Dieta, Resistência Física, Exercícios, Remodelamento Atrial

O consumo prolongado de uma dieta rica em gordura (DRG) causa infiltração de macrófagos no miocárdio, que começa a secretar citocinas envolvidas na inflamação cardíaca. Nesse sentido, a interleucina-1β (IL-1β) desempenha um papel fundamental.^1^ Níveis elevados de IL-1β desencadeiam a produção mitocondrial de espécies reativas de oxigênio (ERO). A explosão de ERO no miocárdio contribui para o desenvolvimento de disfunção diastólica e insuficiência cardíaca com fração de ejeção preservada em modelo experimental de DRG.^1^

Uma revisão sistemática atual realizada por Portes et al.^2^ mostrou que o treinamento de resistência (TR) pode mitigar efeitos nocivos da DRG no coração. O estudo de Portes et al.^2^ revisou artigos originais incluindo roedores com DRG submetidos a um programa de TR de pelo menos quatro semanas comparando animais não treinados. Cinco estudos foram considerados elegíveis, e a subida em escada com carga externa foi o método de TR aplicado.^3 - 7^ Outros três estudos encontraram redução na massa corporal em animais submetidos ao TR,^3 , 5 , 6^ indicando que uma via pela qual o TR pode promover cardioproteção pode ser reduzindo a DRG. No entanto, nenhum desses estudos relatou a ingestão alimentar.^3 , 5 , 6^ Um único estudo realizado por Kim et al.,^4^ avaliou a ingestão alimentar semanal em ratos de meia-idade. Os autores demonstraram que o TR poderia regular positivamente proteínas ligadas à biogênese mitocondrial e reduzir marcadores de estresse do retículo sarcoplasmático sem afetar a ingestão de DRG e a massa corporal.

Quatro estudos avaliaram a massa cardíaca,^4 - 7^ e dois manuscritos indexaram a massa cardíaca pela massa corporal.^4 , 5^ O ajuste da massa cardíaca pela massa corporal permite avaliar a presença de hipertrofia cardíaca não mediada pela massa corporal. O aumento no índice de massa cardíaca foi relatado em um estudo,^5^ enquanto no outro estudo,^4^ nenhuma alteração foi observada após a TR. Lino et al.,^6^ e Melo et al.,^7^ não identificaram maior massa cardíaca não indexada em resposta ao TR. Dado que esses dois estudos observaram, respectivamente, redução e manutenção da massa corporal em resposta ao TR,^6 , 7^ torna-se desafiador assumir, com base no conjunto de quatro estudos que avaliaram a massa cardíaca, se o TR é eficaz na indução de hipertrofia cardíaca. Embora Lino et al.,^6^ tenham fornecido dados sobre o volume semanal de carga realizada no TR, os protocolos de exercício tinham um volume baixo. Esses achados podem apoiar a falta de hipertrofia cardíaca em animais em DRG,^4 , 6 , 7^ o que pode ter ocorrido devido ao volume de TR insuficiente para estimular o crescimento de cardiomiócitos. Assim, maior número de séries, mesmo que resulte em redução da sobrecarga, é uma alternativa viável para promover maior volume de TR.^8^

Todos os cinco estudos consideraram animais machos por serem mais propensos a desenvolver disfunção cardíaca do que fêmeas em modelos de DRG.^3 - 7^ Nesse sentido, 12 semanas de DRG foram associadas ao aumento do volume sistólico final e da pressão sistólica final do ventrículo esquerdo bem como diminuição da massa corporal em camundongos machos, mas não em fêmeas.^9^ É possível hipoteteizar que investigações em animais mais velhos ou períodos mais longos de DRG possam induzir disfunção cardíaca em fêmeas. O desenvolvimento de um protocolo experimental que resulte em disfunção cardíaca ligada a DRG pode ser vantajoso, pois as fêmeas tendem a exibir hipertrofia cardíaca mais significativa em resposta ao treinamento,^10 - 12^ e, ao mesmo tempo, apresentam menor redução na massa corporal.^11 - 13^

Em resumo, o estudo de Portes et al.^2^ descobriu que o TR em animais submetidos à DRG aumenta a biogênese mitocondrial e os marcadores de remodelação tecidual, juntamente com menos inflamação, estresse oxidativo e estresse do retículo endoplasmático. O TR não atenuou a fibrose miocárdica ou preveniu a disfunção dos cardiomiócitos e de proteínas que modulam a cinética do cálcio causado pela DRG.^2^ A principal limitação dos estudos incluídos na revisão sistemática foi a falta de dados sobre os desfechos cardíacos mais importantes, como parâmetros de função sistólica e diastólica do ventrículo esquerdo. Os estudos focaram principalmente em alterações biomoleculares precoces que desencadeiam a disfunção cardíaca causada pela DRG.^3 - 7^ No entanto, é importante notar que essas alterações nem sempre refletem as anormalidades funcionais cardíacas.

**Figura 1:**
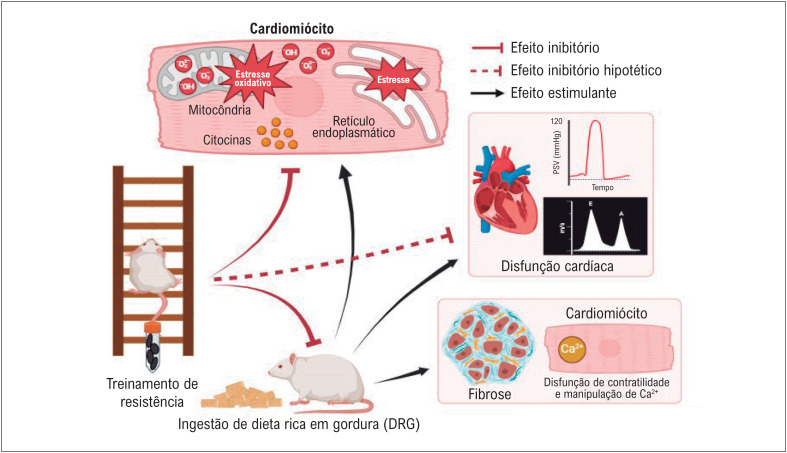
Alterações cardíacas induzidas pela DRG e os efeitos cardioprotetores do TR. O TR pode promover cardioproteção reduzindo indiretamente a ingestão de DRG ou agindo diretamente no nível celular para reduzir o estresse oxidativo, o estresse do retículo endoplasmático e a inflamação cardíaca em roedores. No entanto, a RT não demonstrou atenuar a fibrose ou reverter a disfunção contrátil e os prejuízos na manipulação do cálcio nos cardiomiócitos. Existe a hipótese de que o TR possa reverter a disfunção cardíaca induzida pela DRG, mas não houve estudos que avaliaram parâmetros cardíacos funcionais. Criado por Bio Render.com. PSV: pressão sistólica do ventrículo esquerdo.

À luz de estudos futuros, recomenda-se que as próximas investigações considerem maior volume de treino semanal, seja pelo aumento do número de séries por sessão ou pela frequência de treinamento. Este procedimento possibilitará esclarecer se o TR tem impacto importante na hipertrofia cardíaca em animais sob DRG enquanto clarifica parâmetros de desempenho cardíaco. A Figura 1 abrange os possíveis efeitos benéficos do TR na remodelação cardíaca induzida pela DRG.
