# Supplementary material for: Dieta Rica em Gordura e Coração: Qual é o Real Impacto do Treinamento com Exercícios de Resistência?
Source: Arq Bras Cardiol. 2024 Apr 22;121(4):e20240091. [Article in Portuguese] doi: 10.36660/abc.20240091 (PMC11081092; doi:10.36660/abc.20240091)
Supplement: Translation

# Translation: High-Fat Diet and Heart: What is the Real Impact of Resistance Exercise Training?

**DOI:** 10.36660/abc.20230544i

**Authors:** André Rodrigues Lourenço Dias, Andrey Jorge Serra

**Affiliations:** 1 São Paulo SP Brazil Universidade Federal de São Paulo, Disciplina de Cardiologia, Laboratório de Fisiologia Cardíaca, São Paulo, SP – Brazil

**Keywords:** Heart/physiopatology, Fats/metabolism, Diet, Physical Endurance, Exercises, Atrial Remodeling

Long-term consumption of a high-fat diet (HFD) causes macrophage infiltration in the myocardium, which begins to secrete cytokines involved in cardiac inflammation. In this regard, interleukin-1β (IL-1β) plays a key role.^1^Elevated levels of IL-1β trigger mitochondrial production of reactive oxygen species (ROS). The burst of ROS in the myocardium contributes to the development of diastolic dysfunction and heart failure with preserved ejection fraction in an experimental HFD model.^1^

A current systematic review by Portes et al.^2^has shown that resistance training (RT) can mitigate a variety of harmful effects of HFD on the heart. The article by Portes et al.^2^reviewed original articles carried out in HFD rodents subjected to an RT program of at least four weeks comparing non-trained animals. Five studies were considered eligible, and ladder climbing with external load was the RT method applied.^3 - 7^Another three studies have found a reduction in body mass in animals subjected to RT,^3 , 5 , 6^indicating that one pathway through which RT may promote cardioprotection may be reducing HFD. However, none of these studies reported food intake.^3 , 5 , 6^A single study by Kim et al.^4^assessed weekly food intake in middle-aged rats, who demonstrated that RT could upregulate proteins linked to mitochondrial biogenesis and reduce markers of sarcoplasmic reticulum stress without affecting HFD intake and body mass.

Four studies evaluated cardiac mass,^4 - 7^and two manuscripts have indexed cardiac mass by body mass.^4 , 5^Adjusting cardiac mass for body mass allows for the assessment of the presence of non-body mass-mediated cardiac hypertrophy. An increased index heart mass has been reported for a study,^5^while in the other study,^4^no such change was observed post-RT. Lino et al.^6^and Melo et al.^7^did not identify a larger non-indexed cardiac mass in response to RT. Given that these two studies observed, respectively, a reduction and maintenance in body mass in response to RT,^6 , 7^it becomes challenging to assume, based on the set of four studies evaluating cardiac mass, whether RT is effective in inducing cardiac hypertrophy. Although Lino et al.^6^provided data on the weekly volume of load carried in RT, the exercise protocols were marked by a low volume. These findings may support the lack of cardiac hypertrophy in animals on an HFD,^4 , 6 , 7^which may have been because of an RT volume not sufficient to stimulate cardiomyocyte growth. Thus, a greater number of series, even if this results in a reduction in overload, is a viable alternative to promote a greater RT volume.^8^

All five studies have considered male animals because they are more likely to develop cardiac dysfunction than females in HFD models.^3 - 7^In this regard, 12 weeks of HFD were associated with increased end-systolic volume and end-systolic pressure of the left ventricle as well as decreased body mass in male mice, but not in females.^9^It is possible to hypothesize that investigations in older animals or longer periods of HFD may induce cardiac dysfunction in females. Developing an experimental protocol that leads female rodents exposed to HFD to develop cardiac dysfunction may be advantageous, as females tend to exhibit more significant cardiac hypertrophy in response to training,^10 - 12^while simultaneously showing a lesser reduction in body mass.^11 - 13^

In summary, the study by Portes et al.^2^found that RT in animals subjected to HFD increases mitochondrial biogenesis and tissue remodeling markers, together with less inflammation, oxidative stress, and endoplasmatic reticulum stress. The RT could not attenuate myocardial fibrosis or counteract the impairment in cardiomyocyte function and calcium handling proteins caused by HFD.^2^A main limitation of the studies included in the systematic review was the lack of data on the most important cardiac outcomes, such as left ventricular diastolic and systolic function. The studies mainly focused on the early biomolecular changes that trigger the cardiac dysfunction caused by an HFD.^3 - 7^However, it is important to report that these changes do not always reflect the functional abnormalities at the level of the cardiac organ.

In light of future studies, it is recommended that further investigations consider a higher weekly training volume, either by increasing the number of series per session or by the training frequency. This procedure will clarify whether RT has a major impact on cardiac hypertrophy in HFD animals while investigating parameters of cardiac performance. Figure 1 encompasses the possible beneficial effects of RT on diet-induced cardiac remodeling.

**Figure 1:** HFD-induced alterations in hearts and the cardioprotective effects of RT. The RT may promote cardioprotection by indirectly reducing HFD intake or by directly acting at the cellular level to reduce oxidative stress, endoplasmic reticulum stress, and cardiac inflammation in rodents. However, RT has not been shown to attenuate fibrosis or reverse contractile dysfunction and calcium handling impairments in cardiomyocytes. There is a hypothesis that RT may reverse HFD-induced cardiac dysfunction, but there were no studies that assessed functional cardiac parameters. Created by BioRender.com.
